# Associations between Job Strain and Arterial Stiffness: A Large Survey among Enterprise Employees from Thailand

**DOI:** 10.3390/ijerph15040659

**Published:** 2018-04-02

**Authors:** Orawan Kaewboonchoo, Grace Sembajwe, Jian Li

**Affiliations:** 1 Faculty of Public Health, Mahidol University, Bangkok 10400, Thailand; orawan.kae@mahidol.ac.th; 2 Department of Environmental, Occupational and Geospatial Health Sciences, CUNY Institute for Implementation Sciences in Population Health, CUNY Graduate School of Public Health and Health Policy, New York, NY 10027, USA; grace.sembajwe@sph.cuny.edu; 3 Institute of Occupational, Social and Environmental Medicine, Centre for Health and Society, Faculty of Medicine, University of Düsseldorf, Düsseldorf 40225, Germany

**Keywords:** job strain, cardiovascular risk, employees, gender, Thailand

## Abstract

As an intermediate endpoint to cardiovascular disease, arterial stiffness has received much attention recently. So far, the research on work stress and arterial stiffness is still sparse and inconsistent, and no investigations on work stress and cardiovascular health among the Thai working population have been reported. Therefore, we conducted an epidemiological study among 2141 Thai enterprise employees (858 men and 1283 women) who were free from any diagnosed cardiovascular disease. Work stress was measured using Karasek’s Job Demand–Control model for job strain (a combination of high demand and low control). Arterial stiffness was evaluated by a non-invasive approach using pulse-wave analysis based on a finger photoplethysmogram. Multivariable linear regression was applied to examine associations between job strain and arterial stiffness. In men, job strain was significantly associated with arterial stiffness (β  =  0.078, 95% confidence interval  =  0.026 to 0.130), after accounting for sociodemographic, behavioral, dietary and biomedical factors. However, the association in women was not significant. As the first study in Thailand on work stress and cardiovascular risk, we found that job strain might be an important risk factor for cardiovascular disease among Thai working men. Further studies with longitudinal design are warranted.

## 1. Introduction

As the leading cause of mortality, cardiovascular disease (primarily coronary health disease and stroke) accounts for approximately one third of all deaths worldwide. In recent years, much evidence has highlighted the role of work stress in the etiology of cardiovascular disease [1]. It is estimated that work stress is associated with a 10–40% higher risk of developing cardiovascular disease [2]. Karasek’s Job Demand–Control model, which postulates that job strain results from the joint effects of high job demand and low job control [3], has been widely used in work stress epidemiological research. For example, a large collaboration that included 13–14 cohort studies, among nearly 200,000 workers, revealed that job strain increased the risk of coronary heart disease by 23% [4] and of ischemic stroke by 24% [5].

When focusing on early prevention strategies for stress-related cardiovascular disease at work, it is important to gain some insight into the different stages of the cardiovascular disease process. In 1996, Kasl recommended that the influence of work stress should be investigated at intermediate cardiovascular endpoints before the manifestation of disease [6]. As a well-established early pathological indicator of cardiovascular disease [7,8], atherosclerosis (an obstructive condition resulting from the accumulation of lipid and calcium deposits) has received notable attention since then. In the past two decades, over a dozen epidemiological studies have been conducted, indicating that work stress resulted in an increased risk of atherosclerosis [9]. Clinically, in addition to atherosclerosis there is another medical condition, arterial stiffness, which refers to medial degeneration due to vascular remodeling and loss of arterial elasticity [10]. Increasingly, new findings also suggest arterial stiffness is tightly linked to cardiovascular disease [11,12,13]. To the best of our knowledge, six cross-sectional studies examined the potential associations between work stress and arterial stiffness, four from Japan [14,15,16,17], one from Taiwan [18] and one from The Netherlands [19]. Four of them found significant positive associations, i.e., higher work stress was related to increased arterial stiffness [15,16,17,19] whereas one study reported null findings [18]; and another study discovered significant negative associations [14]. Furthermore, the results between men and women were not consistent. Otsuka et al., identified meaningful relationships between work stress and arterial stiffness in women only [17], but other studies confirmed the findings in men [15,16,19]. As a result, more research is needed in other populations and settings.

Thailand is an interesting case. First, although morbidity and mortality rates from cardiovascular disease have been mostly declining in advanced industrialized nations, in Thailand these rates have been rising notably over the past few decades [20]. For example, from 2011 to 2015 the annual mortality rates in Thailand increased from 22.5 to 29.9 per 100,000 due to coronary heart disease and from 30.0 to 43.3 per 100,000 due to stroke [21]. In addition, as a traditional agricultural society, the major occupational health problems in Thailand were due to physical, chemical and biological hazards [22]. Under rapid economic growth, the proportion of industrial and service sectors has dramatically increased from less than 30% in the 1980s to more than 60% in 2010 [23]; accordingly, psychosocial stress in the workplace is regarded as an emerging occupational hazard in Thailand. In a recent survey of Thai workers, 51% reported that they had to work very fast and 13% declared they had no decision-making power at work [24]. Finally, some studies have been conducted in Thailand to investigate the associations of work stress with mental and behavioral outcomes [24,25]. However, while the research on cardiovascular epidemiology in Thailand is still restricted to traditional risk factors, such as smoking and hypertension [26], to date there is no research evidence available on the effects of work stress on cardiovascular health among the Thai working population. Therefore, the aim of this current study was to examine the association of work stress, measured as job strain, with arterial stiffness in a large sample of Thai enterprise employees. Given the mixed findings between men and women on work stress and cardiovascular disease in studies elsewhere [27,28], for this investigation we also explored gender differences.

## 2. Materials and Methods

### 2.1. Sample

Due to limited research evidence and mixed findings on work stress and arterial stiffness (see details in Introduction), we were not able to extract solid parameter estimates for the associations. In view of the previous six cross-sectional studies on this topic, the sample sizes varied from 300 to 4000. Therefore, we projected that a sample size with 2000–3000 subjects would be appropriate for our study. This cross-sectional study was conducted with 19 different enterprises located in Bangkok and five other provinces in Thailand (Chiang Mai, Nakhon Ratchasima, Rayong, Ayutthaya and Trang) representing different geographic and socioeconomic regions in this country. The 19 enterprises were from a wide range of industrial sectors including canned food, snack food, cooking oil, footwear, pharmaceutical, ceramics, toy, steel, petrochemical and auto parts. All the 2935 employees in these enterprises were invited to a comprehensive health check comprising a questionnaire and a medical examination (detailed below). In total, 2218 employees (76% of 2935 invitees) took part in the health check. Due to voluntary participation, some employees declined the medical examination, yielding 2208 employees who had complete data on both questionnaire and medical examination (99% of 2218 respondents). The subjects with missing values on medical data (particularly arterial stiffness) did not differ significantly from the participants with complete data regarding sociodemographic or work-related characteristics. Furthermore, subjects with any diagnosis or medication related to cardiovascular disease were excluded from the current analysis (*n* = 67). The final sample size of this study consisted of 2141 subjects (858 males and 1283 females). All participants gave written informed consent. This study protocol was approved by the Ethics Review Committee of Mahidol University, Bangkok, Thailand (No.: 23/2561).

### 2.2. Measurements

#### 2.2.1. Job Strain

Job strain was assessed using Karasek’s Job Content Questionnaire [29], whose validated Thai version has been made available [30]. In our current study, two scales were used: namely, demand with 5 items, and control with 9 items. Items were scored using a four-point Likert scale (1 = strongly disagree, 4 = strongly agree). The score range for demand was 12–48 and for control it was 24–96, according to pre-defined scale formulations [29]. Higher scores indicated higher demand and control. Cronbach’s α coefficients for the demand scale and the control scale were 0.65 and 0.73, respectively. Following the theory of the Job Demand–Control model [3,29], a median split of total sample or sub-sample gender-specific scale values was used to construct four combinations of demand and control, the so-called quadrant approach, i.e., low strain (low demand + high control), active (high demand + high control), passive (low demand + low control), and high strain (high demand + low control).

#### 2.2.2. Arterial Stiffness

Arterial stiffness was evaluated by a non-invasive approach, pulse-wave analysis based on the second derivative of the finger photoplethysmogram (FCP-3166, Fukuda Denshi, Tokyo, Japan). Each subject was asked to rest at least 10 min before starting the examination, which required participants to be in a supine position with records taken from the second fingertip of the left hand while it was at the same height as the subject’s heart. The temperature in the testing room was kept at 23–25 °C. The waveform produced consisted of four systolic waves (a, b, c and d waves) and one diastolic wave (e wave). The a and b waves corresponded to the early systolic phase, the c and d waves corresponded to the late systolic phase, and the e wave corresponded to the diastolic phase of the second derivative of the finger photoplethysmogram. The amplitude levels of the a, b, c, d and e waves from the baseline were recorded, with the values above the baseline being positive and those below it negative. Then, the aging index was calculated by using the ratios of (b–c–d–e)/a. This index represented arterial stiffness, with higher values indicating lower central and peripheral arterial elasticity [31], which has been shown to be related to cardiovascular risk [32,33].

#### 2.2.3. Covariates

In addition, data on sociodemographic factors (age, gender, marital status, education and employment position), behavioral factors (smoking, alcohol drinking and physical exercise), dietary factors (vegetable intake and fatty food intake) and biomedical factors (body mass index (BMI) and hypertension) were also collected through the questionnaires and medical examinations.

### 2.3. Statistical Analysis

All analyses were conducted using the SAS 9.2 (SAS Institute, Cary, NC, USA). Firstly, descriptive statistics were generated. Means and standard deviations (SDs) were investigated for continuous variables and relative frequencies were examined for categorical variables. We applied Student’s *t* test (for continuous variables) or the chi-square test (for categorical variables) to compare the differences between men and women. Normality was examined by the Kolmogorov–Smirnov test and all the continuous variables in our study followed normal distribution. Secondly, univariable associations between work stress and arterial stiffness were tested. Pearson correlation coefficients among demand, control and stiffness were calculated for the total sample, for men, and for women. Furthermore, the differences in arterial stiffness across four job-strain groups were also tested by analysis of variance. Finally, multivariable linear regression was applied for associations between job strain and arterial stiffness (dependent variable). In addition to the four job-strain groups, the single dimensions, i.e., continuous measures of demand and control (expressed as an increase or decrease per SD), were also used in regression modeling. Again, the regression analyses were performed in the total sample, for men, and for women. The results are shown as β coefficients and 95% confidence intervals (CIs), together with *R*^2^. Several regression models were calculated with incremental adjustments for sociodemographic, behavioral, dietary and biomedical factors.

## 3. Results

Table 1 shows the characteristics of the study sample (*n* = 2141). Forty percent of the study participants were men (*n* = 858). The mean age of men and women was similar at 34 years and approximately two-thirds of them were married. Both education and employment position levels were much lower in women than in men. The lifestyle behaviors were relatively healthy in Thai female employees, who very seldom smoked or drank alcohol, engaged more in physical exercise; and 60% of women had vegetable intake daily. More than 30% of male employees were categorized as being overweight and obese and nearly 20% were hypertensive. Women reported significantly higher demand and lower control, resulting in higher levels of job strain than men. Also, the index of arterial stiffness was harder in women compared with men (*p* < 0.0001).

The univariable results revealed that, in the total sample, both demand and control were significantly correlated with arterial stiffness (Table 2). Accordingly, the high-strain group had the highest score of arterial stiffness while the low-strain group had the lowest score (Table 3). The results of sub-group analyses according to gender were not significant, but the univariable associations were stronger among men than women.

Table 4 presents the multivariable regression findings for the total sample. Although the statistical level of significance was not reached, trends in increasing arterial stiffness with separate dimensions (i.e., continuous high demand and low control) or combined construct (i.e., four job strain groups) were demonstrated in the first model (adjusted for age, gender, marital status and socioeconomic status). The strength of the associations remained unchanged when taking further covariates into account (models II–IV). The adjusted *R*^2^ was slightly improved from 0.324 (model I) to 0.333 (model IV). Using the fully adjusted model, we found that, in men, high demand was significantly associated with arterial stiffness; moreover, high job strain in terms of high demand in combination with low control exerted the strongest effect on arterial stiffness (β  =  0.078, 95% CI  =  0.026 to 0.130). In women, no significant associations of job strain with arterial stiffness were observed (Table 5).

## 4. Discussion

The aim of our study was to examine the associations between work stress (based on the Job Demand–Control model) and arterial stiffness on stress-related cardiovascular disorders at work in Thailand, in the first investigation of this kind. The findings are in line with cross-sectional evidence from Japan and The Netherlands [15,16,17,19], preliminarily showing that job strain in terms of high demand and low control was associated with arterial stiffness, particularly in male employees, and that the associations could not be attributed to other known risk factors for cardiovascular disease, such as age, marital status, socioeconomic status, lifestyle behaviors, vegetable and fatty-food consumption, body mass index and hypertension. We also conducted sensitivity analyses to test the stability of our findings. For instance, in addition to the quadrant approach, we also used the quotient approach for job strain defined as the ratio between demand and control (balanced by item weighting) [34,35]; in addition to the continuous measures of demand, control and demand/control (D/C) ratio, these measures were also divided by tertiles into three groups (high, intermediate and low). All these sensitivity analyses exhibited similar patterns to what we present in the Results section (details are available upon request).

It is important to mention another related intermediate cardiovascular disease endpoint, atherosclerosis, with similar global findings, although most of the evidence has stemmed from studies in Europe and America, with only few conducted in Asia, (Japan [36] and China [37,38]). Clinically, the coronary artery and carotid artery are often used to evaluate atherosclerosis. By using techniques of coronary angiography or computed tomography scanning, four [36,39,40,41] out of six studies [36,37,39,40,41,42] found work stress was not related to coronary atherosclerosis. However, the findings derived from ultrasonographic carotid intima-media thickness have been more consistent. To date, 16 reports based on 11 studies in total are available [38,43,44,45,46,47,48,49,50,51,52,53,54,55,56,57]. Among them, 12 reports based on 8 studies asserted significantly positive associations [38,43,44,46,47,48,49,50,51,52,53,55], 3 studies reported null findings [45,56,57], whereas 1 study observed significantly negative associations [54]. It should be highlighted that several longitudinal studies have confirmed the significant contribution of work stress to progression of carotid artery atherosclerosis, such as the 4-year longitudinal study from Denmark [53], the Cardiovascular Risk in Young Finns Study with 6-year follow-up [52], the Kuopio Ischemic Heart Disease Risk Factor Study in Finland with 4-year follow-up [43,44] and the Pittsburgh Healthy Heart Project in the USA with 3- and 6-year follow-ups [49,50]; however, significant associations at baseline disappeared during the 9-year follow-up in the Multi-Ethnic Study of Atherosclerosis from the USA [56]. When considering gender differences, the findings are mixed. Three studies did not find significant associations in both men and women [45,56,57], but one study found significant associations in both genders [53]. While three studies discovered significant associations in women only [38,42,47] and six significant reports in men only [43,44,46,49,51,52]; null associations in men were also identified by one study [36].

In this Thai study, we found a positive association between work stress and arterial stiffness only in men, but not in women. For a long time, it has been observed that in the labor market women encounter worse psychosocial working conditions and experience higher stress levels [58], which is supported by our study, too. On the other hand, the gender differences in occurrence and development of cardiovascular disease do exist. In women, it is less common during the premenopausal period and then sharply rises after menopause [59]. Such differences are found not only in the manifested cardiovascular diseases, but also in the early course of the asymptomatic stage, such as arterial stiffness and atherosclerosis [60,61]. Therefore, the pattern of gender differences with regard to the effect of work stress on cardiovascular disease is not clear [27,28]. Some recent research has suggested gender as an effect modifier of the association between work stress and health [62]. Furthermore, in psychosocial stress research, particularly among women, it has been suggested that working women are under dual stress from work and from family. Stress related to family and household might confound the associations between work stress and health for women. For example, the UK Whitehall II study found stress at home predicted coronary heart disease in women, but not in men [63]; the Stockholm Female Coronary Angiography Study indicated that women exposed to both work and family stress suffered most from accelerating atherosclerosis [42]; and a recent study using data from the Health and Retirement Study in the USA reported that the highest mortality group came from women who had work stress together with adverse family circumstances [64]. Although no obvious evidence from cohort research is available in Asia, some preliminary cross-sectional studies did demonstrate the burden of dual stress among Asian women on health, such as hypertension and menstrual disorders [65,66]. Thus, several global organizations including the European Society of Cardiology (Sophia Antipolis, France) and the American Heart Association (Dallas, TX, USA) have expressed concerns that further research on the broader psychosocial environment is warranted [67,68].

When looking more closely at Table 4 and Table 5, it is clear that the effects driven by job strain were primarily explained by demand. Cultural differences should be considered. Qualitative research suggests that low job control is usually perceived as a stressor at work in Western societies with individualistic values. By contrast, in societies with collectivistic values, such as Asia, high control would be perceived as a distressing lack of structure [69]. Therefore, it is not surprising to see that job control did not show any effect on arterial stiffness [15,18], or even that harmful effect was observed [14] in some Asian studies. In addition, the Job Demand–Control model was applied to measure work stress in most of the epidemiological studies on work stress and arterial stiffness/atherosclerosis mentioned above. Alternatively, another work stress model in terms of Effort–Reward Imbalance developed by Siegrist is also worthy of noting. This model emphasizes the harmful effects of failed reciprocity between effort spent at work and reward received in turn (high effort/low reward) [70]. In the past two decades, cumulative research has figured out that effort–reward imbalance at work is associated with an elevated risk for cardiovascular disease independently of job strain [71,72]. Interestingly, a couple of studies revealed that effort–reward imbalance is significantly related to atherosclerosis cross-sectionally and progression of atherosclerosis longitudinally, either in Eastern culture or in Western culture, not only in men but also in women [37,38,43,53].

One novel approach of our study is the measurement of arterial stiffness. As “the non-invasive gold standard for measuring arterial stiffness”, pulse-wave velocity has been well recognized and accepted clinically [73]. In general, carotid-femoral or brachial-ankle pulse-wave velocity are recommended by the European Network for Non-invasive Investigation of Large Arteries and the American Heart Association (Dallas, TX, USA) [74,75], as applied by those epidemiological studies on work stress and arterial stiffness [14,15,16,17,18,19]. Technically, pulse-wave velocity could be measured in any defined segment of the human circulation or from a single-point measurement. Therefore, a new method of digital finger photoplethysmography has been developed in recent years to measure pulse-wave velocity [76,77] and the aging index derived from this new method has been verified to have a high repeatability and best correlations with other standard measures of pulse-wave velocity in clinical settings [78], while epidemiological studies also indicate the utility of finger photoplethysmography as an index of arterial stiffness predicting cardiovascular morbidity and mortality [32,33]. With respect to stress and arterial stiffness measured by finger photoplethysmography, one fascinating lab study found that stress-induced (mental arithmetics and cold pressor test) changes in pressure pulse reflection by fingertip signals were associated with blood flow changes in brachial and femoral arteries measured by ultrasound [79]. As far as we know, our study is the first epidemiological study worldwide examining relationship between psychosocial stress and pulse waveform from finger, with the novelty of non-invasiveness, inexpensiveness and convenience, especially for large-scale epidemiological research. Certainly, more studies are warranted in the future to justify its applicability. One obvious advantage of using clinically validated measure of arterial stiffness is to avoid the potential bias of self-reported psychosocial exposure assessment caused by disease perception, since all individuals with manifest cardiovascular disease were excluded from the analyses and the remaining ones were asymptomatic.

Despite these merits, several limitations to our study need to be addressed and must be taken into consideration when interpreting our findings. First, as the study design is cross-sectional, it is impossible to draw any causal inference for the direction of the observed associations. Yet, given the evidence of several longitudinal studies in this field [43,44,49,50,52,53], the findings of our study seem to be in accordance with the mainstream research, particularly in men. Second, much evidence has suggested that other psychosocial factors, such as depression and anxiety, play important roles in the etiology of cardiovascular disease [80,81]. One earlier study observed that the association between work stress and arterial stiffness was partly mediated by depressive and anxiety symptoms [19]. Moreover, genetic factors, such as Neuregulin-1 genotype and catechol-o-methyltransferase gene polymorphism, were found to have moderating effects between work stress and atherosclerosis [82,83]. Nonetheless, these factors were not included in our study. The third limitation is the restricted study sample. Although a wide range of industries and regions across Thailand were included, we were not able to recruit a nationally representative sample. Thus, the power of generalization based on current findings is limited. Also, the sample size was based upon an estimation from previous cross-sectional studies and not from a direct sample-size calculation.

## 5. Conclusions

In conclusion, despite these limitations, this study adds an important new piece of evidence, that job strain is associated with arterial stiffness in Thai male employees. Extended studies by longitudinal investigations and intervention trials are needed to promote a healthy work environment.

## Figures and Tables

**Table 1:** Characteristics of study subjects (*n* = 2141).

Variables		Men (*n* = 858)	Women (*n* = 1283)	*p*-Value
Age (years)		34.346 ± 7.945	34.885 ± 8.492	0.1346
Marital status	Single (unmarried, divorced, or widowed)	294 (34.27%)	432 (33.67%)	0.7758
	Married	564 (65.73%)	851 (66.33%)	
Education	College or below	674 (78.55%)	1071 (83.48%)	0.0041
	University or above	184 (21.45%)	212 (16.52%)	
Employment position	Low (without managerial responsibility)	581 (67.72%)	1058 (82.46%)	<0.0001
	High (with managerial responsibility)	277 (32.28%)	225 (17.54%)	
Smoking	Yes	279 (32.52%)	7 (0.55%)	<0.0001
	No	579 (67.48%)	1276 (99.45%)	
Alcohol drinking	Yes	193 (22.49%)	17 (1.33%)	<0.0001
	No	665 (77.51%)	1266 (98.67%)	
Physical exercise	Yes	223 (25.99%)	545 (42.48%)	<0.0001
	No	635 (74.01%)	738 (57.52%)	
Vegetable intake	Seldom or sometimes	497 (57.93%)	515 (40.14%)	<0.0001
	Everyday	361 (42.07%)	768 (59.86%)	
Fatty food intake	Seldom or sometimes	713 (83.10%)	1051 (81.92%)	0.4813
	Everyday	145 (16.90%)	232 (18.08%)	
Body mass index (BMI)	Obese (BMI > 30)	42 (4.90%)	102 (7.95%)	0.0008
	Overweight (BMI > 25 and < 30)	226 (26.34%)	268 (20.89%)	
	Normal (BMI < 25)	590 (68.76%)	913 (71.16%)	
Hypertension	No	693 (80.77%)	1139 (88.78%)	<0.0001
	Yes	165 (19.23%)	144 (11.22%)	
Demand		33.871 ± 5.655	35.128 ± 6.188	<0.0001
Control		73.608 ± 10.897	69.230 ± 11.795	<0.0001
Demand/control (D/C) ratio		0.941 ± 0.216	1.046 ± 0.279	<0.0001
Arterial stiffness index		−0.637 ± 0.292	−0.442 ± 0.330	<0.0001

Continuous variables are expressed as mean ± standard deviation (SD) and categorical variables are expressed as number (percent). Statistical analysis was performed with Student’s *t* test for continuous variables and chi-square test for categorical variables.

**Table 2:** Correlations among demand, control and arterial stiffness index (correlation coefficients).

Study Samples		Demand	Control	Arterial Stiffness Index
Total (*n* = 2141)	Demand	1		
	Control	0.060 **	1	
	Arterial stiffness index	0.066 **	−0.057 **	1
Men (*n* = 858)	Demand	1		
	Control	0.093 **	1	
	Arterial stiffness index	0.059	−0.007	1
Women (*n* = 1283)	Demand	1		
	Control	0.074 **	1	
	Arterial stiffness index	0.026	−0.003	1

Pearson correlation, ** *p* < 0.01.

**Table 3:** Differences of arterial stiffness index by job strain groups (means and SDs).

Variables		Total (*n* = 2141)	Men (*n* = 858)	Women (*n* = 1283)
Job strain	Low strain	−0.559 ± 0.324	−0.664 ± 0.298	−0.450 ± 0.322
	Active	−0.532 ± 0.326	−0.643 ± 0.288	−0.432 ± 0.330
	Passive	−0.518 ± 0.333	−0.644 ± 0.296	−0.447 ± 0.340
	High strain	−0.484 ± 0.331	−0.604 ± 0.285	−0.434 ± 0.329
	*p* value	0.0034	0.1839	0.8850

Analysis of variance.

**Table 4:** Associations of job strain with arterial stiffness in total sample (*n* = 2141, β coefficients with 95% CIs).

Variables		Model I	Model II	Model III	Model IV
Demand	Increase per SD	0.011 (−0.000, 0.023)	0.012 (−0.000, 0.023)	0.011 (−0.001, 0.023)	0.011 (−0.001, 0.022)
Control	Decrease per SD	0.007 (−0.006, 0.019)	0.006 (−0.006, 0.018)	0.006 (−0.006, 0.018)	0.007 (−0.005, 0.020)
	Adjusted *R*^2^	0.324	0.328	0.328	0.333
Job strain	Low strain	0	0	0	0
	Active	0.011 (−0.023, 0.045)	0.011 (−0.023, 0.045)	0.012 (−0.022, 0.046)	0.010 (−0.024, 0.044)
	Passive	0.009 (−0.029, 0.047)	0.008 (−0.030, 0.046)	0.010 (−0.028, 0.048)	0.011 (−0.027, 0.049)
	High strain	0.034 (−0.002, 0.070)	0.033 (−0.002, 0.069)	0.034 (−0.002, 0.070)	0.034 (−0.001, 0.070)
	Adjusted *R*^2^	0.324	0.328	0.328	0.333

Linear regression. Model I: Adjusted for age, gender, marital status, education and employment position; Model II: Model I + additionally adjusted for smoking, alcohol drinking and physical exercise; Model III: Model II + additionally adjusted for vegetable intake and fatty food intake; Model IV: Model III + additionally adjusted for BMI and hypertension.

**Table 5:** Associations of job strain with arterial stiffness in men (*n* = 858) and women (*n* = 1283) (β coefficients with 95% confidence intervals (CIs)).

Variables		Men (Fully Adjusted Model)	Women (Fully Adjusted Model)
Demand	Increase per SD	0.020 (0.002, 0.037) *	0.006 (−0.009, 0.022)
Control	Decrease per SD	0.015 (−0.004, 0.033)	0.002 (−0.014, 0.019)
	Adjusted *R*^2^	0.230	0.307
Job strain	Low strain	0	0
	Active	0.027 (−0.022, 0.076)	0.015 (−0.029, 0.059)
	Passive	0.036 (−0.018, 0.090)	0.000 (−0.041, 0.041)
	High strain	0.078 (0.026, 0.130) **	0.015 (−0.032, 0.062)
	Adjusted *R*^2^	0.233	0.308

Linear regression, * *p* < 0.05, ** *p* < 0.01, adjusted for age, marital status, education, employment position, smoking, alcohol drinking, physical exercise, vegetable intake, fatty food intake, BMI and hypertension.
